# The Effect of Sport-Specific Brain Endurance Training on Performance in Elite Orienteering Athletes

**DOI:** 10.3390/sports14010032

**Published:** 2026-01-08

**Authors:** Kenneth Buch, Richard Thomas, Walter Staiano, Simon Lønbro

**Affiliations:** 1 Department of Public Health, Section for Sports Science, Aarhus University, 8000 Aarhus C, Denmark; loenbro@ph.au.dk; 2 Team Danmark, 2605 Brøndby, Denmark; rith@teamdanmark.dk; 3 Department of Physical Education and Sport, University of Valencia, 46000 Valencia, Spain; walterstaiano@gmail.com; 4 Department of Psychology, Biological and Cognitive Psychology, University of Southern Denmark, 5230 Odense N, Denmark

**Keywords:** brain endurance training, cognitive training, elite orienteering, aerobic training

## Abstract

Aim: To investigate the effect of a 6-week sport-specific BET intervention on cognitive and physical performance in elite orienteering athletes. Methods: A single-arm cross-over study with an initial 6-week control period (CON) followed by a 6-week brain endurance training (BET). Thirteen Danish national team orienteering athletes participated in the study. CON athletes adhered to planned physical, cognitive, and technical training. BET athletes added 20 min of route choice assessment (RCA) training after each weekly aerobic training session. The 30 min Stroop color-word task and a sport-specific RCA task evaluated general and sport-specific cognitive performance. A submaximal (1000 m) and a maximal (5000 m) running test were also conducted. Endpoints were assessed pre and post CON and post BET. Results: Average time used per RCA task was 1.4 ± 0.4 s lower following BET (27%) (*p* = 0.009) compared with no change after CON. Similarly, the total number of correct Stroop answers increased by 13.8 ± 5.21 points (2%) after BET with no change after CON. RCA time use declined steeply from session 1–7, whereafter average time use plateaued. Running performance did not differ significantly between periods. Conclusion: BET improved sport-specific performance and aspects of general cognitive performance, and may effectively improve cognitive parts important for elite orienteering performance.

## 1. Introduction

Elite orienteering is a complex endurance sport demanding concurrent interplay between physical and cognitive factors [1]. While performing at the limit of their physical ability on distances varying from 2 to 21 km, athletes are constantly cognitively challenged. These cognitive challenges comprise complex decision-making, anticipation, and simplification of information. The cognitive processes are pivotal, especially when making route choices and simultaneously translating the map and terrain [2]. Athlete experience and competitive pressure significantly modulate the nervous system’s response to orienteering’s dual demands. Experienced athletes may require less effort to solve complex spatial tasks than novices, mitigating mental strain. Conversely, high competitive stress may exacerbate cognitive load, leading to elevated autonomic arousal and negatively influencing decision-making [3].

The implications of mental fatigue (MF) on both cognitive and physical performance are topics of great interest for both the scientific community [4,5] as well as coaches and athletes [6]. Mental fatigue has been defined as a psychobiological state caused by prolonged demanding cognitive activities [7]. It has been shown that MF inhibits performance by either increased time use in maximal running performance at a fixed distance [8,9], reduced time to exhaustion in submaximal cycling performance [10], or lower pace setting in intermittent running performance [11]. In addition, MF may negatively affect cognitive performance, for example by inhibiting marksmanship decision-making skills [12] and soccer players’ ability to choose the right passing strategy [13].

Studies showing an association between both training state [14] and resilience to MF in elite cyclists, as well as performance in cognitive tasks [15] collectively hypothesize that training the brain’s resilience to MF would improve performance [16]. Consequently, the concept of brain endurance training (BET) that integrates aerobic endurance training and cognitive training has been developed. The rationale behind BET is that compared with physical training alone, systematically exposing the athletes to MF in combination with physical training over time will lead to better performance [16]. Marcora et al. [16] used a 12-week BET regime comprising three weekly one-hour sessions with moderate training improved time to exhaustion performance, with lower RPE levels in moderately trained males. Similar findings have been shown to improve agility test performance in professional football players [17,18], handgrip performance [19,20], and time trials in well-trained and elite cyclists [21]. Studies also report improved cognitive performance following BET intervention, i.e., less time used to solve response inhibition tasks and a sport-specific cognitive task [16,19,20,21].

Cognitive training is increasingly used to enhance athletes’ decision-making and executive functions. Targeted cognitive exercises yield measurable improvements in skills such as anticipation. Systematic reviews confirm this training positively influences elite athletes’ decision-making in controlled settings. While the transfer to actual competition is often modest, evidence supports that training the brain—using sport-specific drills (e.g., map memory for orienteers)—is crucial for developing the mental edge needed in high-level competition [22,23]. To increase the potential of BET in a specific sport it has been proposed that sport-specific BET is required [24]; however, earlier BET studies have not used this approach. Using sport-specific tasks in a sports context would arguably improve both sport-specific performance and expand the resilience to MF. In orienteering the decision-making process during route choice assessment can serve this purpose. Lam et al. [25] supports the idea that accumulated assessment of route choices can lead to MF in orienteering athletes.

Acknowledging the simultaneous high demands of cognitive and physical skills in elite orienteering, it is plausible that a BET intervention would have a positive impact on these skills. However, no studies on orienteering athletes have been published, and whether the findings mentioned above from other athlete populations transfer to orienteering remains to be investigated.

Thus, the present study aims to investigate the effect of a 6-week sport-specific BET intervention on cognitive and physical performance in elite orienteering athletes. Cognitive performance is assessed using both a sport-specific test and a general test. Maximal and submaximal physical performance, as well as objective and subjective physiological load, during these tests are included.

We hypothesized that BET, compared with the control period, would improve performance significantly in both the sport-specific and general cognitive tests. Furthermore, we hypothesized that BET would improve maximal and submaximal physical performance significantly either by reducing time use in maximal performance or the physiological load experienced during the tests.

## 2. Materials and Methods

### 2.1. Design and Participants

The inclusion criteria were that the participants were senior (above 18 years of age) male and female orienteering athletes from the Danish national team squad. Athletes were excluded if they sustained an injury that prevented completion of training or testing, or they chose to withdraw themselves. All athletes were informed verbally and in writing by the investigators about all aspects of the study and formally agreed to take part by signing a written consent that could be retracted with no further consequences for the athletes.

**Figure 1:**
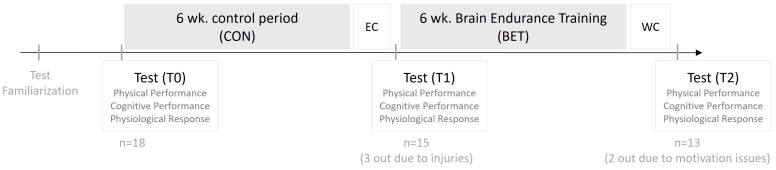
Study design and flow of participants through the study. EC: European Championships; WC: World Championships. Physical and cognitive tests were performed at baseline (T0) after test familiarization, after EC (T1) and after WC (T2).

In a single-arm cross-over design (Figure 1) participants acted as their own controls. Following a test familiarization and a test day (T0) with physical and cognitive performance tests, all athletes completed the control period (CON) comprising 6 weeks of usual training leading up to the European Championships. After a similar test day (T1), the 6-week BET intervention period leading up to the World Championships followed, after which all athletes completed the final test day (T2).

### 2.2. The CON Period

In CON, athletes adhered to all physical, cognitive, and technical training that was planned and managed by the national team coaches.

### 2.3. BET Intervention

The six weeks of BET included three weekly sessions comprising the already planned physically demanding aerobic training sessions (high-intensity aerobic training (HIT) sessions) to which approximately 20 min of specific route choice assessment (RCA) training was added. In the BET period athletes adhered to all physical, cognitive, and technical training that was planned and managed by the national team coaches.

The total amount of physical training, including secondary training (i.e., strength training, biking etc.), technical training, and isolated cognitive technical training (technical desk training, i.e., the RCA training of the BET) during both CON and BET was logged for each athlete (via Sportlyzer.com). Physical training was categorized into low (LIT), moderate (MIT), and high intensity (HIT) using individual HR. HIT sessions encompassed zone 3, 4, and 5 intensities with HR ranging from 82.5 to 100% of individual HR max. Sessions were conducted as prolonged endurance, interval-based, and competition running on all surfaces with and without orienteering-specific activities.

The RCA part of the BET was conducted using software specifically developed for this project. The RCA software (version 1.3) for training comprised more than 2000 unique orienteering legs designed so that different route choices could be assessed. Firstly, an orienteering leg map was shown; the time started; the athletes then decided their route choice as quickly and accurately as possible; and the time stopped. Then, in a new image, all possible route solutions were displayed, and the athletes marked the route they decided on. See Supplementary Material, Figure S1A+B for examples of orienteering legs from the RCA software. Finally, before moving on to the next leg, athletes received feedback on their choice as either “optimal”, “ok”, or “poor”. The scoring system is explained below in “Sport-specific cognitive test”. In a few instances, more than one option could be the “optimal” or “ok” response.

### 2.4. Endpoint Evaluation

The athletes completed the physical and cognitive performance tests on three different days: (T0) before CON, (T1) after CON, and (T2) after BET. On each day and in the same order the athletes completed a 1000 m submaximal running test, a 5000 m maximal running test, a general cognitive test, and a sport-specific cognitive test. To quantify fatigue and subjective training load, and to exclude bias from psychological parameters, psychological measurements were conducted before and after all four tests (motivation, mood, and experienced workload—see Supplementary Materials for detailed description and results). The participants were told to do light training the day before each test, eat, drink, and sleep well, and avoid any out-of-the-ordinary disturbances in their daily lives. Before the submaximal running test athletes completed a 20 min self-instructed low-pace warm-up.

### 2.5. Physical Performance

Before the physical tests, body weight (kg) and height (cm) were measured using standard equipment (Tanita T6360, Tanita Corporation, Tokyo, Japan). Submaximal self-paced (males @ 3:40 and females @ 4:10 min/km) endurance performance was assessed on a 1000 m track and a field test completed on a standard 400 m outdoor athletics track. Whistle signals for each 100 m ensured athletes kept to the right speed. After the submaximal test, a 5000 m maximal test was conducted. Total time as well as 400 m lap times were measured using a photocell system (Microgate Witty, Microgate, Italy). Average and maximal heart rate were monitored and registered throughout both physical tests with a wearable heart rate monitor (Polar M430 watch with H10 heart rate monitor, Polar, Finland). Blood lactate concentration was measured before the physical tests, after the submaximal test, and again one and three minutes after the completion of the maximal performance. Fingertip blood samples were collected into a capillary tube (1 mL), stored, and analyzed the same day for lactate concentration (Biosen C-line, EKF-diagnostic GmbH, Barleben, Germany). On a Borg scale from 6 to 20, athletes rated their Perceived Exertion (RPE) after the submaximal test and after the maximal running performances [26].

### 2.6. Cognitive Performance

#### 2.6.1. General Cognitive Test (Response Inhibition Task)

A 30 min Stroop [14,27] color–word task evaluated general cognitive performance using E-Prime 3 (Psychology Software Tools Inc., Pittsburgh, PA, USA). Athletes responded as quickly and accurately as possible to colored words displayed by pressing correspondingly colored keys on the keyboard. They matched the randomly computer-chosen ink color to the word meaning (incongruent), except for the color red, where they matched the word’s actual meaning. If the ink color was blue, green, or yellow, participants pressed the key corresponding to the ink color, creating an incongruent condition. If the ink color was red, they pressed the key corresponding to the word’s meaning, creating a congruent condition. Thirty practice attempts were allowed before the inhibition task to ensure that the participant understood the concept fully. Visual feedback was given after each word in the form of correct or incorrect answer, response speed, and accuracy score. Outcome measures were reaction time as well as accuracy score (as a percentage of correct responses in relation to the total) and total number of correct responses.

#### 2.6.2. Sport-Specific Cognitive Test

Sport-specific cognitive performance was assessed using the same software as the RCA training of the BET but comprised 100 new and unique orienteering legs. Participants were instructed to respond as quickly and accurately as possible. Outcome measures were average response time (ms) per leg and accuracy score (total number of and percent correct answers). Scores for each response were allocated by an expert coach based on an analysis of world class performance over time and according to the following criteria: 2 points = an optimal route choice within the time gap of 0–2 s; 1 point = an achievable (“OK”) route choice leading to a 3–7 s time loss; 0 points = a poor decision leading to an ≥8 s time loss. A maximum of 200 points was possible. The scoring system was inspired by [28], where participants scored from zero to two points for each attempt.

### 2.7. Athlete-Reported Psychological Factors in Relation to Testing

Before and after all physical and cognitive tests, athletes answered questionnaires on self-perceived mood using the Brunel Mood Scale (BRUMS) [29], subjective workload using The National Aeronautics and Space Administration Task Load Index (NASA-TLX) rating scale [30], and motivation using three different questions relating to general motivation (how motivated do you feel right now?), readiness (how ready do you feel right now?), and focus (how focused do you feel right now?). The detailed methodology and results from the questionnaires are presented in Supplementary Materials, Table S1 due to the word limit of the manuscript.

## 3. Statistics

To our knowledge, this is the first study to investigate the effect of BET on physical and cognitive performance in orienteering athletes and thus data on expected changes are unavailable for a precise sample size calculation. Unpublished pilot study data collected in 2020 (unpublished) indicated that a similar BET period improved the number of “optimal” responses of a 20 min RCA from 60–70 “optimal” responses with variation of approximately 7. Using these data in a sample size calculation (Stata 17, Stata Corp LLC, Lakeway, TX, USA) with 80% statistical power, a 5% level of significance, and an expected 10% drop-out provided a sample size of 19 participants needed for the present investigation. This sample size is comparable with previous studies in other athlete populations and in studies on the effect of mental fatigue on performance [4,5,16,17,19].

To investigate the effect of the BET intervention, the differences in changes in primary and secondary endpoints during the control condition and the BET intervention are analyzed using an ANOVA with repeated measures. For post hoc analyses, Student’s paired *t*-tests were used. All analyses were performed in Stata 17 (Stata Corp LLC, TX, USA). Data are presented as mean values ± standard error of the mean (SEM).

## 4. Results

Eighteen Danish national team orienteering athletes volunteered to participate in the study (23 ± 3 years of age, 14 ± 4 years of participation, 4 ± 2 years at international level): eight females (VO^2^max = 57.8 ± 7.3 mL × kg^−1^ × min^−1^, height = 174 ± 7 cm, weight = 62.9 ± 8.9 kg) and ten males (VO^2^max = 69.5 ± 10.0 mL × kg^−1^ × min^−1^, height = 184 ± 7 cm, weight = 71.2 ± 7.1 kg). During CON three athletes did not complete the study due to injuries and during BET two dropped out because of motivational issues. Thus, 13 athletes completed the study (Figure 1).

The athletes performed equal volumes of total HIT training in the CON and BET periods. CON completed a total of 02:22 ± 00:20 h:min per week, compared with 02:20 ± 00:18 h:min per week in BET. BET comprised an average 17.3 ± 1.0 sessions during the six-week period. Thus, the average HIT session prior to the BET lasted 49 min. The RCA part of the session lasted on average 23:19 ± 8:59 min:s. and the athletes’ average perceived mental load of the RCA part during all sessions was 5.9 ± 2.1 on the 1–10 BRUMS scale. The average time spent per RCA task decreased over time (see Supplementary Materials, Figure S2) leading to a progression in number of tasks conducted during the duration of the RCA part. Furthermore, in total volumes of training in the CON and BET periods there was no disparity in LIT volume and technical training. The BET group performed significantly (*p* < 0.05) lower volumes of MIT (48 min) and strength training (18 min) but a significantly higher (*p* > 0.05) volume of technical desk training (1:19 h, due to the BET intervention). Ultimately, BET showed significantly lower total training volume compared to CON (10:36 ± 00:46 vs. 09:00 ± 00:48 h:min).

### 4.1. Cognitive Performance

**Figure 2:**
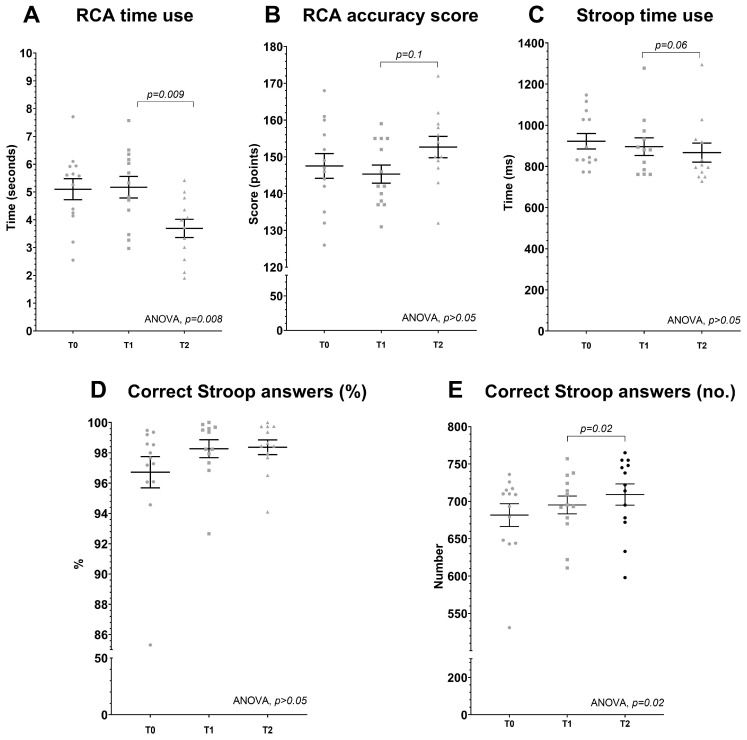
(**A**–**E**) Route choice assessment and Stroop test. Average time in seconds spent during each RCA assignment (2A) and total score in the RCA test (2B) at baseline (Test 0), after the CON period (Test 1) and after the BET period (Test 2). Average time in seconds spent during each Stroop test (2C) and percent correct answers in the Stroop test (2D) at the same time points. Individual data presented in grey circles, squares, and triangles. Mean data presented as mean ± SEM. Specific *p*-value are presented from the one-way ANOVA and Student’s *T*-test with the latter denoting statistical differences between time points.

According to the ANOVA analysis, time used per RCA task changed significantly between time points (*p* = 0.008, 95% CI: −0.2; −1.2 s) (Figure 2A). The average time used per RCA task was significantly reduced by 1.4 ± 0.4 s (27%) (*p* = 0.009, 95% CI 0.4; 2.4 s) after BET from 5.2 ± 0.3 s at T1 and 3.7 ± 0.4 s at T2. There was no difference between T0 (5.1 ± 1.4 s) and T1 following CON. The ANOVA analysis revealed no significant changes between T0, T1, and T2 in RCA test score (*p* > 0.05); however, a numerical 6.2 ± 3.5 (4%) increase from 146.5 ± 2.4 points to 152.7 ± 2.9 points (*p* = 0.1, 95% CI: −1.6; 13.9 points) was observed after BET from T1 to T2 (Figure 2B).

The total number of correct answers in the Stroop test differed significantly between time points according to ANOVA (*p* = 0.02). The total number of correct answers increased by 13.8 ± 5.2 points (2%) after BET from 695.3 at T1 to 709.1 at T2 (*p* = 0.02; 95% CI: 2.5; 25.2) with no significant change after CON from T0 to T1 (*p* = 0.2, 95% CI: −35.0; 7.7) (Figure 2E). The percentage of correct Stroop answers was not significantly different between T0 (96.7 ± 1.0%), T1 (98.3 ± 0.5%), and T2 (98.4 ± 0.3 %) (ANOVA: *p* > 0.05) (Figure 2D). Furthermore, no changes between T0, T1, and T2 were found in either congruent or incongruent conditions (*p* > 0.05). Despite a numerical decrease, there was no significant difference between T0 (922 ± 37 ms), T1 (896 ± 43 ms), and T2 (867 ± 47 ms) in average time use per Stroop task (ANOVA: *p* > 0.05) (Figure 2C). Following BET, from T1 to T2, average time use per Stroop task decreased numerically by 29 ± 13 ms (*p* = 0.06, 95% CI: −58; 1 ms).

**Figure 3:**
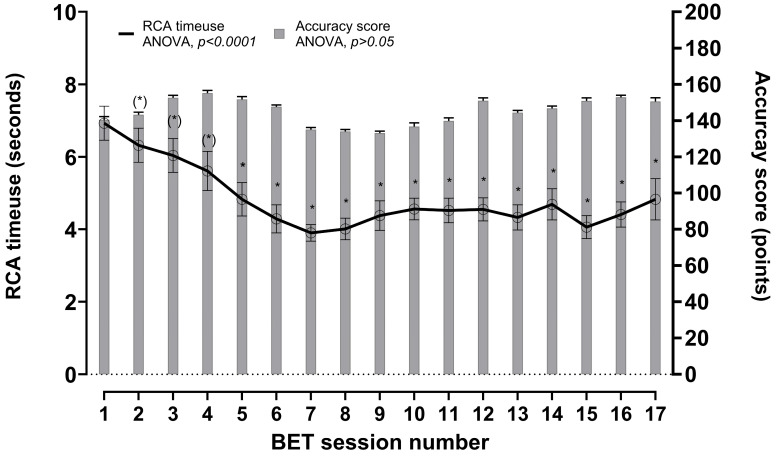
Development in RCA time use (seconds) and accuracy score (number of correct answers) during the 17 BET sessions. * denotes significant (*p* < 0.05) difference in time use compared with first BET session analyzed by Student’s *T*-tests as post-hoc test following a one-way ANOVA. (*) denotes *p*-values between 0.05–0.1.

The average time used per single RCA task over the 17 BET sessions changed significantly (ANOVA *p* < 0.0001; 95% CI: −0.1; −0.2 s), and as indicated in Figure 3 there was a steeper decline in average time use from session 1–7, whereafter average time use seemed to stagnate from session 9 onwards.

The RCA score did not change over the 17 BET sessions (ANOVA *p* > 0.05), indicating that the quality of RCA answers was unchanged despite the initial faster response time.

### 4.2. Physical Performance

According to the ANOVA analyses, there were no significant differences between T0, T1, or T2 in any of the physical performance test or physiological endpoints. Thus, there were no significant differences in either physical performance or associated assessments of RPE and blood lactate concentration. See Table 1 for specific results from all physical and physiological evaluations.

## 5. Discussion

This study aimed to investigate the effects of a six-week BET intervention on cognitive and physical performance of elite orienteering athletes. The main findings were that adding a demanding sport-specific cognitive training load immediately after the high-intensity physical training sessions in elite orienteering athletes improved the sport-specific cognitive performance and also partly general cognitive performance. Specifically, time use in a specific route choice assessment task was reduced while maintaining the response quality. In addition, the improved RCA time use was primarily achieved in the initial seven BET sessions, whereafter average time use stagnated. General cognitive performance measured as total number of correct answers (but not as percentage of correct answers) in the Stroop test improved significantly following BET. Physical performance and physiological measures were not affected by BET.

### 5.1. BET and Sport-Specific Cognitive Performance

In line with our hypothesis, sport-specific cognitive performance improved significantly. When presented with a route choice, athletes used on average 1.4 s less in each route choice decision (Figure 2A) with no change in response quality (Figure 2B). This is interesting because of the salience of the decision-making process in the elite orienteering performance [2]. In orienteering competitions, with an average of 20 route choices, our finding suggests that an elite athlete would be able to reduce their total time by 28 s, which is considered a highly relevant improvement. Improving cognitive performance through better decision-making is very likely to be even more orienteering performance enhancing if athletes either spend the “saved time” on other cognitive demands such as anticipation and simplification [2], to keep a better flow in the competition, or improve running biomechanics by not having to hold the map in a visible reading position [31]. We cannot explain the mechanisms behind the improved sport-specific cognitive performance or the scope of the possible orienteering performance enhancement. We can speculate that the improvement in response times while maintaining accuracy score may be explained by the athletes adapting to the cognitive load during the initial sessions (Figure 3), after which the results suggest a ceiling effect with regards to further improvements in response speed. From a skill learning perspective, the faster response times in the RCA could be a consequence of a task-specific learning effect. However, despite the fact that the route choices and format were very familiar to the athletes, the structure and systematized approach of the RCA was new to them and may have reduced the task-specific learning effect. Part of this improvement could be the development of a more efficient route choice decision approach. This could be explained by a more efficient visual search behavior as training with the RCA progressed; however, we have no data supporting this and future research could include investigations using eye tracking to investigate this.

### 5.2. BET and General Cognitive Performance

The total number of correct Stroop answers improved significantly following BET and not CON (Figure 2E). This is in line with previous findings [16,17,19,20,21]. Also, average Stroop time use was reduced numerically (*p* = 0.06) following BET (Figure 2C), and as shown earlier the response accuracy score was unchanged (Figure 2D). In general, the Stroop scores were above 96%, showing a high level in elite orienteering athletes in this cognitive task, but the high test scores also form a ceiling effect, largely limiting improvement in the test. The cognitive demands in the response inhibitory task and in orienteering performance are closely connected, suggesting that there was a transfer effect using the specific RCA during training and the generic response inhibition performance observed in the Stroop scores during testing.

### 5.3. BET and Physical Performance

In contrast to our hypothesis, physical performance did not change. Earlier studies show that BET may lower the RPE during activity and/or maximize endurance performance [16,17,19,20,21]. Recent studies, however, showed no difference in physical performance after 6 weeks of BET training [32]. The COVID-19 pandemic excluded the use of laboratory tests and outdoor field tests with reduced precision were the only option. The project was conducted from March to June under different weather conditions and temperatures. Specifically, at the time of T2 a heatwave affected most athletes during testing, and this likely reduced their physical performance and explains the diverging findings compared with previous studies.

### 5.4. Strengths and Limitations

A significant strength of the present study is that it investigates the effect of a sport-specific, combined cognitive and physical training intervention in elite athletes in the prime of their competitive season before two major competitions. This rare opportunity enables insight into the effect and feasibility of a specific intervention under real-life conditions, improving the transferability of the findings and the relevance of the findings from a practitioner’s point of view, and thus reducing the gap between scientific research and practical implementation [33,34].

The single arm cross-over design was chosen to enable inclusion of as many athletes as possible since a two-armed randomized controlled design would have to allocate half of the athletes to a control group. Including a control group using a general response inhibition task during the intervention was not possible due to the number of eligible athletes.

As mentioned previously, a sport-specific intervention and test approach in the present study is likely needed [24]; however, this also poses the question of whether the effect of the BET intervention is affected by the similarity between the intervention and test. All orienteering athletes in the present study were used to route choice tasks, and creating a relevant but still unfamiliar test would be unrealistic and also undermine the sport-specific approach. The structure and systematized approach of the RCA program were new to all athletes and may reduce the potential learning effect. The RCA test was developed specifically for the present study and has not been validated scientifically, but on the other hand the authenticity of the route choice program in relation to the demands of the sport is considered to improve the relevance of the test and thus the practical transferability of the findings. Unfortunately, the only estimation of reliability of the RCA test that can be deduced from our data is the observation that the CON period did not elicit any significant difference in average response time (0.07 ± 0.34 s). Another possibility investigated in the literature is to use generalized cognitive tasks which are individualized and adapted to the level of fatigue of athletes [35]. 

Despite the considerations behind the sample size of the present study and the resemblance with similar studies in elite athletes, the relatively low number of athletes warrants further research involving strategies to limit the implications of mental fatigue in elite endurance athletes and orienteering athletes in particular.

## 6. Conclusions

Elite orienteering athletes showed improved sport-specific and partially general cognitive performance after six weeks of BET. Physical performance and physiological parameters, however, remained unaffected. In summary, and with respect to the small sample size, this is the first study in elite orienteering athletes to provide evidence that a pragmatic and sport-specific BET could be a valuable training tool for enhancing specific and general cognitive aspects of performance.

### Practical Implications

- The present study provides an original and effective training method for developing cognitive performance in elite orienteering athletes.
- A sport-specific intervention is needed to improve relevant cognitive aspects of the specific sport but may also improve aspects of general cognitive function.
- A six-week intervention period prior to peak orienteering performance proved effective.

## Figures and Tables

**Table 1:** Results from the physical performance tests performed at baseline (Test 0), after the CON period (Test 1) and after the BET period (Test 2). No significant differences were observed. Data presented as mean ± SEM.

	TEST 0	TEST 1	TEST 2
1000 m submax % HR	87.7 ± 0.9	85.0 ± 1.1	87.2 ± 1.8
1000 m submax RPE	12.2 ± 0.6	10.8 ± 0.9	12.2 ± 0.7
1000 m submax lactate level (mmol/L)	2.2 ± 0.2	1.6 ± 0.2	2.1 ± 0.2
5000 m time (min:ss)	17:37 ± 01:55	17:11 ± 01:50	18:13 ± 02:21
5000 m average % HR max	95.8 ± 0.6	95.2 ± 0.5	95.4 ± 0.9
5000 m lactate level (mmol/L)	10.0 ± 0.5	9.4 ± 1.0	9.5 ± 0.4
5000 m RPE	18.5 ± 0.2	18.5 ± 0.2	18.3 ± 0.3

## Data Availability

Due to EU General Data Protection Regulation original data cannot be shared.

## Supplementary Materials

The following supporting information can be downloaded at: https://www.mdpi.com/article/10.3390/sports14010032/s1, Table S1: Results from the psychological questionnaires; Figure S1A+B: Screen shots from RCA program; Figure S2: RCA Development during BET.
