# Supplementary material for: The Effect of Sport-Specific Brain Endurance Training on Performance in Elite Orienteering Athletes
Source: Sports (Basel). 2026 Jan 8;14(1):32. doi: 10.3390/sports14010032 (PMC12845864; doi:10.3390/sports14010032)
Supplement: Supplementary file 1 [file sports-14-00032-s001.zip › sports-4029836-supplementary.pdf]

Supplementary material

Psychological questionnaires

All questionnaires were completed on a tablet and stored online in an encrypted system.

Mood

The Brunel Mood Scale (BRUMS) is used to assess mood. This questionnaire, which is based on the Profile of Mood States, contains 24 items (e.g., angry, uncertain, miserable, tired, nervous, energetic) divided into six respective subscales: anger, confusion, depression, fatigue, tension, and vigour. The items are answered on a 5-point Likert scale (0 not at all, 1 a little, 2 moderately, 3 quite a bit, 4 extremely), and each subscale, with four relevant items, can achieve a raw score in the range of 0 to 16 with a higher number indicating a stronger expression. Because of the particular interest in mental fatigue in the present study, we specify that an alteration in the subscales for fatigue and vigor is an indication of mental fatigue (Cutsem et al., 2017).

Subjective workload

The National Aeronautics and Space Administration Task Load Index (NASA-TLX) rating scale (Hart and Staveland 1988) assessed subjective workload. It is composed of six subscales: mental demand (How mentally demanding was the training/test?), physical demand (How physically demanding was the training/test?), temporal demand (How much time pressure did you feel during the training/test?), performance (How successful do you think you were?), effort (How hard did you have to work to accomplish your level of performance?) and frustration (How irritating was the training/test?). The participants score on each of the items on a scale divided into 20 equal intervals anchored by a bipolar descriptor (e.g. high/low). This score is multiplied by 5, resulting in a final score between 0 and 100 for each of the subscales with a higher number indicating a higher workload. In this study due to technological issues the participants scored on a scale divided into 10 intervals. The score was then multiplied with 10 instead of 5.

Motivation

Motivation was measured through three different subjective questions embracing a state of a general motivation. Motivation (how motivated do you feel right now?), readiness (how ready do you feel right now?), focus (how focused do you feel right now?) The participants scored on a scale from 1 to 10, where a higher number indicated a high feeling.

Table S1 summarizes the results from the psychological measurements collected during the tests. No significant differences between T0, T1 or T2 were found. The motivation was at its lowest at T2, while the experience of the cognitive load from the Stroop and RCA-test was in general experienced lower at T2.

Table S1 - Results from psychological questionnaires

n=13	Test 0	Test 1	Test 2
Motivation for the physical tests (Scale 1-10)	7.2±0.5	6.8±0.6	5.3±0.6
Motivation for Stroop colour word task (Scale 1-10)	7.8±0.5	8.0±0.5	6.1±0.8(*)
Subjective training load of the Stroop test “how cognitively demanding was the task?” (NASA-TLX)	72.3±5.0	75.0±4.8	71.7±7.1
Changes in fatigue after Stroop (BRUMS)	-1.8±0.6	-0.7±1.4	-0.2±0.7
Changes in vigour after Stroop (BRUMS)	-0.9±0.5	-1.1±1.9	-0.4±0.3
Motivation for RCA-test (scale 1-10)	6.6±0.5	6.7±0.4	5.7±0.7
Subjective training load of the RCA-test “how cognitively demanding was the task?” (NASA-TLX)	74.6±4.8	70.8±3.8	62.5±7.9
Changes in fatigue after RCA (BRUMS)	2.0±0.6	2.4±1.1	-0.2±1.6
Changes in vigour after RCA (BRUMS)	-1.3±0.6	0.92±0.8*	0.3±0.5

Table S1. Results from the psychological questionnaires during the tests performed at baseline (Test 0), after the CON period (Test 1) and after the BET period (Test 2). * denotes significant difference compared with the previous test ($p<0.05$) analysed by the Wilcoxon signed-rank test due to non-parametric data. Data presented as mean±SEM.

Figure S1A+B - Screen shots from RCA program

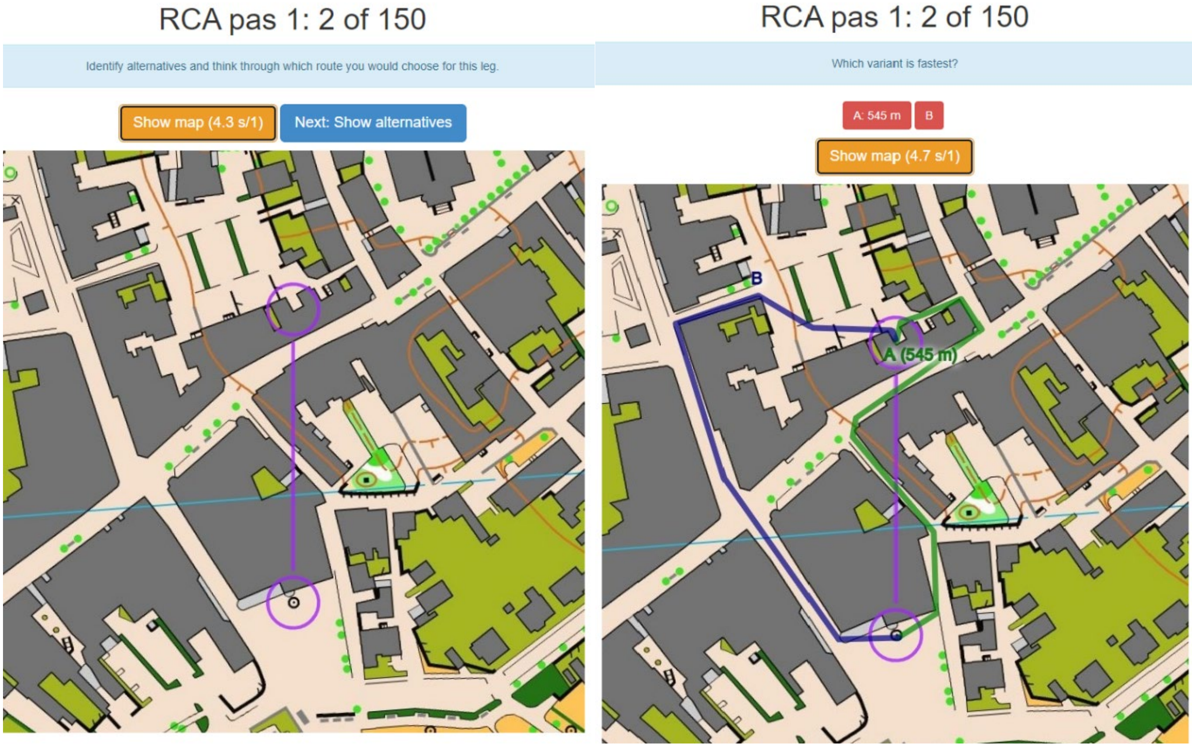

Figure S1A+B shows the display on the computer screen during the RCA-program.

Figure S2 – RCA development during BET

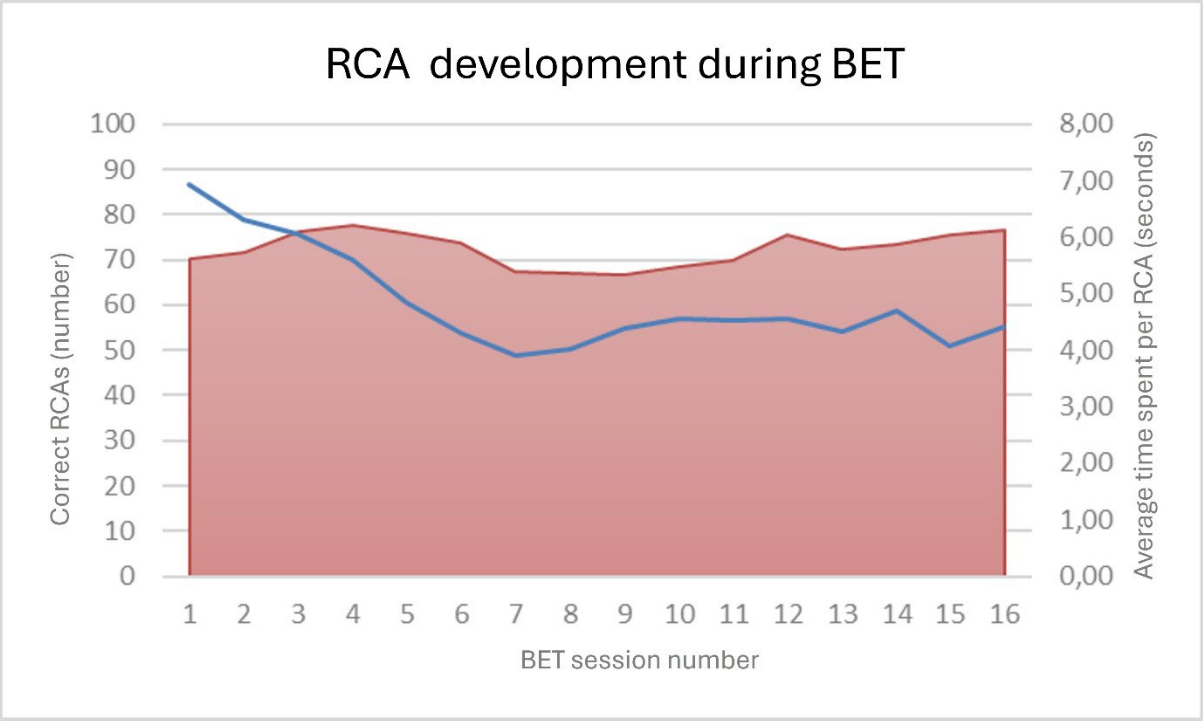

Figure S2 shows RCA development over time.
